# Exploring wild *Aspleniaceae* ferns as safety sources of polyphenols: The case of *Asplenium trichomanes* L. and *Ceterach officinarum* Willd.

**DOI:** 10.3389/fnut.2022.994215

**Published:** 2022-09-12

**Authors:** Adrià Farràs, Montserrat Mitjans, Filippo Maggi, Giovanni Caprioli, María Pilar Vinardell, Víctor López

**Affiliations:** ^1^Department of Biochemistry and Physiology, Faculty of Pharmacy and Food Sciences, Universitat de Barcelona, Barcelona, Spain; ^2^Department of Pharmacy, Faculty of Health Sciences, Universidad San Jorge, Zaragoza, Spain; ^3^School of Pharmacy, Università di Camerino, Camerino, Italy; ^4^Instituto Agroalimentario de Aragón-IA2, CITA-Universidad de Zaragoza, Zaragoza, Spain

**Keywords:** bioeconomy, cytoprotection, cytotoxicty, ethnopharmacology, ferns, functional food, polyphenolic phytochemicals

## Abstract

The forest ecosystem is a source of material resources used since ancient times by mankind. Ferns are part of different oriental systems of traditional medicine due to the phytochemical variety of their fronds, which have allowed their traditional use to be validated through ethnopharmacological studies. In Europe, different cultures have used the same fern with a wide variety of applications due to its presence in most European forests. In recent years, studies on the phytocharacterization and biological activity of the fronds of the main European ferns have been published. In this study, the presence of polyphenolic phytochemicals has been evaluated by high-performance liquid chromatography-tandem mass spectrometry (HPLC-MS/MS) in the fronds of two wild ferns together with *in vitro* activities in non-tumoral and human tumoral cell lines. The polyphenols were extracted from *Asplenium trichomanes* L. and *Ceterach officinarum* Willd. by cold maceration using methanol. The main phytochemicals of polyphenolic origin in the extracts of *A. trichomanes* and *C. officinarum* determined by HPLC-MS/MS were the flavonol hyperoside and the phenolic acid chlorogenic acid, respectively. This different polyphenolic nature of both extracts contributes to the divergence of the behavior experienced in the biological activities tested, but none of the extracts showed a cytotoxic or phototoxic profile in the different tested cell lines. However, the cytoprotective values in front of the H_2_O_2_ oxidative stress induced in the 3T3 and HaCaT cell lines position these extracts as possible candidates for future health applications.

## Introduction

Currently, in most countries, forest management is oriented to obtain wood resources. However, in the last decade, there has been an increase in the demand for natural food production, and the global food demand for 2050 is predicted to triplicate the actual demand. Forests may be one via to ensure the food and nutritional security of the population according to the World Food Security (1), especially in industrialized societies due to its lower relationship with nature than rural societies. Bioeconomy is the discipline that covers all activities related to the production, use, and transformation of bioresources, and their functions and principles, among which we find food production, animal feed, and the generation of products, energy, and services (2). Currently, the European Commission’s concept of the bioeconomy incorporates sustainability and circularity with the aim of being respectful with the environment. The food plant resource is one of the main branches of innovations in the bioeconomy due to the broad spectrum of macronutrients and micronutrients in plants compared to foods from animal sources (3).

*Pteridophytes* (from Greek *pteron*, meaning “wing” and *phyton*, meaning “plants”) are the tracheophyta plants with an authentic development of the vascular system and that reproduces sexually by spores (4). Currently, the potential uses of ferns are underestimated as only the properties of a small number of the around 13,000 species of ferns have been studied (5). In 2017, Cao et al. (6) published a review of the valuable reservoir of phytochemicals with the corresponding properties, among which antioxidant, anticancer, antimicrobial, and anti-inflammatory activities have been demonstrated.

Literature reviewing the uses of ferns is irregular worldwide. The continents with the highest number of records in decreasing order are Asia-Tropical, South America, and Asia-Temperate. Nevertheless, Asia-Tropical has approximately three times more reviewed literature uses of ferns than South America (7). Ferns are currently considered a source of polar phytochemicals as phenolic acids and flavonoids with therapeutic and nutritional value in eastern countries, especially in Nepal, India, and China (8–10). This is the case of some ferns described in the traditional Chinese medicine, as the case of *Pteridium aquilinum* (L.) Kuhn (*Dennstaedtiaceae*) (10). In addition, the presence of non-polar phytochemicals, such as fatty acids and lipids, has also been described in ferns (11).

Because of the limitations of the floral bioindicator methodology of Ellenberg values, the need for new ecological bioindicators is derived (12). Ferns are susceptible to little environmental changes, and this taxon is positioning itself as an important ecological indicator, as reported in an ecological indicator study in Mexico (13). In Europe, one of the most important families of ferns is the *Aspleniaceae* family (14), particularly at the Mediterranean coast characterized by its moderated temperatures and high humidity (15). The phenotype studies classified as *Aspleniaceae* family (from Greek *a*, meaning “without” and *splen*, derivate to “spleen” in reference to its use to treat ailments of that organ), known as “spleenwort,” in the eupolypods II group (16, 17). The sporophytes of this taxon are reported in different forms, but they have the common character to be epipetric ferns. Because they grow in rock habits they are also popularly known as “rock ferns”. Nowadays, in some areas of Europe, the *Pteridophytes* distribution has been characterized as the case of Sicily (18). In Spain, researchers have determined the distribution of Iberian Peninsula ferns (19), that is, the case of the *Banco de Datos de Biodiversidad de Cataluña* (20), being the *Asplenium trichomanes* L. (*Aspleniaceae*) (21) and *Ceterach officinarum* Willd. (*Aspleniaceae*) (22) the predominant fern species in the Prades mountains (Tarragona, Spain). *Asplenium ceterach* L. is a synonym name of *Ceterach officinarum* Willd.

**IMAGE 1:**
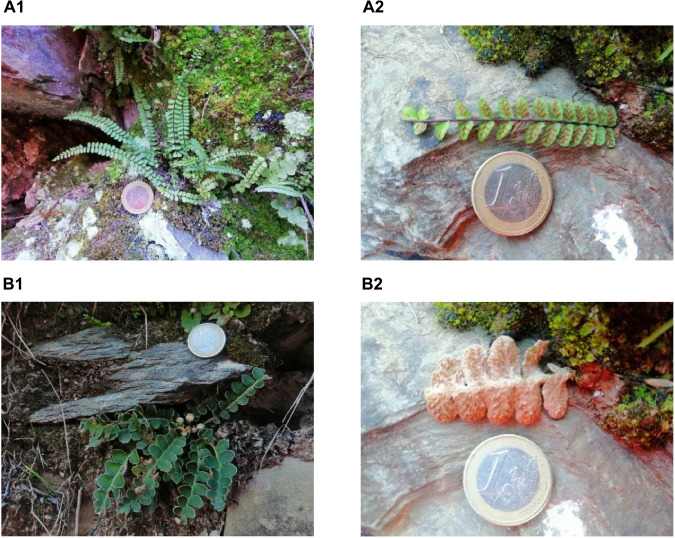
Face fronds (1) and underside frond (2) of fresh *Asplenium trichomanes* L. (*Aspleniaceae*) **(A)** and fresh *Ceterach officinarum* Willd. (*Aspleniaceae*) **(B)** by Adrià Farràs at Prades mountains. The euro coin referents the dimension of the frond **(A2, B2)**.

The sporophyte is the principal characteristic part in the identification taxonomy of ferns (23). The *A. trichomanes* and *C. officinarum* fronds present different morphological features that facilitate the difference between both species, as shown in Image 1. The frond of *A. trichomanes* has a long black petiole with parallel oval pinnas not directly united with the rachis. While the frond of *C. officinarum* has a short petiole with the pinnas attached directly to the rachis alternately forming a saw. In addition, the soris of *C. officinarum* has the characteristic of being covered with reddish scales on the underside frond (24). Through the ambivalence of the Latin names used in the past text and the different names attributed to the same plant in different regions, *A. trichomanes* has been sometimes confused with another *Aspleniaceae* ferns as *C. officinarum* (16).

In Catalonia, the popular name of *A. trichomanes* is *falzia vermella* and for *C. officinarum* is *dauradella* (24). In English literature, these ferns are popularly known as “maidenhair spleenwort” for *A. trichomanes* (25) and “golden herb” for *C. officinarum* (26). Previous studies have considered the fronds of *A. trichomanes* and *C. officinarum* as reservoirs of flavonols (kaempferol and its glycosides) (27) and cinnamic acids (as chlorogenic acid) (28), respectively. The fact that leads to position these ferns as valuable resources of polyphenolic phytochemicals for nutritional applications. The reported traditional uses of *A. trichomanes* and *C. officinarum* in Pyrenees have been used for respiratory tract diseases. The fronds tisane from both ferns has been used for its anticatarrhal properties and specifically *A. trichomanes* as an antitussive (29, 30). These ethnopharmacological studies highlight the presence of phytochemicals in both species of these two active ferns against respiratory tract infectious diseases. Positioning both ferns as possible functional foods in the prevention of colds (31). Other described uses of the fronds of *A. trichomanes* are for the treatment of alopecia and kidney stones (16). In the case of the fronds of *C. officinarum*, in Catalonia, it has also been described as a vasotonic (32) in *Alt Empordà* and for the treatment of cold sores in the Prades mountains (33).

In this article prior to the study of the *in vitro* activity against situations of oxidative stress of the methanolic extracts of *A. trichomanes* fronds (ATM) and *C. officinarum* fronds (COM) harvested in the Prades mountains, we have evaluated the cytotoxicity and phytocharacterization of both extracts.

## Materials and methods

### Chemicals and reagents

Sigma-Aldrich (Milan, Italy) served 36 analytical standards (which were gallic acid, neochlorogenic acid, chlorogenic acid, p-hydroxybenzoic acid, 3-hydroxybenzoic acid, caffeic acid, vanillic acid, syringic acid, p-coumaric acid, ferulic acid, 3,5-dicaffeoylquinic acid, ellagic acid, delphinidin-3,5-diglucoside, delphinidin-3-galactoside, cyanidin-3-glucoside, petunidin-3-glucoside, pelargonidin-3-rutinoside, pelargonidin-3-glucoside, malvidin-3-galactoside, rutin, isoquercitrin, quercitrin, myricetin, isorhamnetin, hyperoside, kaempferol, catechin, epicatechin, procyanidin B2, procyanidin A2, phloridzin, phloretin, hesperidin, naringin, resveratrol, and trans-cinnamic acid) of the 38 phytochemicals, determined by high-performance liquid chromatography-tandem mass spectrometry (HPLC-MS/MS). While kaempferol-3-glucoside and quercetin were served by PhytoLab (Vestenbergsgreuth, Germany). The 1,000 mg/mL concentration was used for each stock solution. In which the pure references substance was dissolved by HPLC-grade methanol and then stored at 5°C in glass vials until the evaluation. The HPLC-grade methanol was also used to prepare the diluting stock solutions for the working solutions. The HPLC-grade methanol and formic acid (99% concentration) was informed by Sigma-Aldrich and Merck, respectively. A 0.2 μm polyamide filters, which were used to filter all liquids, were administered by Sartorius Stedim provided (Goettingen, Germany). Before injecting into the HPLC, all samples were filtered by Phenex™ RC 4 mm 0.2 μm syringeless filters (Phenomenex, located in Castel Maggiore, BO, Italy). The ultrapure water (resistivity of >18 MΩ cm) was obtained by A Milli-Q SP Reagent Water System (Millipore, Bedford, MA, United States). The coolant trypan blue (0.4%) dye was obtained and was expedited from Sigma-Aldrich (Madrid, Spain). The reactive viability thiazolyl blue tetrazolium bromide (MTT), dimethyl sulfoxide (DMSO) solvent, the marker 2,7-dichlorodihydrofluorescein diacetate (DCF), and photosensibility chlorpromazine hydrochloride (CPZ, CAS No. 69-09-0) also were supplied by Sigma-Aldrich. The products to maintain proper cell growth except for the HyClone fetal bovine serum (FBS) were purchased from Thermo Scientific (Northumberland, United Kingdom). These products were Dulbecco’s modified Eagle’s medium (DMEM) with and without phenol red, phosphate-buffered saline (PBS), L-glutamine solution (200 mM), and penicillin–streptomycin solution (10,000 U/mL penicillin and 10 mg/mL streptomycin) were supplied by Lonza (Verviers, Belgium). This distributor also provides the trypsin-ethylenediaminetetraacetic acid (EDTA) solution (170,000 U/L trypsin and 0.2 g/L EDTA). The TPP (Trasadingen, Switzerland) supplemented the 75 cm^2^ culture flasks and 96-well plates.

### Plant material

After we verified that *A. trichomanes* L. and *C. officinarum* Willd. were located at Prades mountains 41°17′34″N 1°02′42″E geographical coordinates (Tarragona, Spain) by *Banco de Datos de Biodiversidad de Cataluña* (20), the fronds of these ferns were harvested from this area. We obtained a dried voucher stored at Herbarium of Universidad San Jorge (Zaragoza, Spain), *A. trichomanes* L. with the voucher n° 005-2016 and *C. officinarum* Willd. with the voucher n° 006-2016. These ferns were authenticated by Dr. J.A. Vicente Orellana from Universidad CEU San Pablo (Madrid, Spain) using botanical keys.

### Preparation of methanolic extract of *Asplenium trichomanes* L. and *Ceterach officinarum* Willd. fronds

Once we obtained the dried powdered fronds, these parts were macerated with methanol for 24 h at room temperature. The result was a methanolic extract which was filtered by Whatman N°4 filter paper and evaporated using a rotatory evaporator with a thermostatic bath at 30°C to eliminate the solvent. This process was repeated three times until exhaustion of the plant material. This process is described by us in a previous publication (34). The extracts were kept at −20°C until experimental bioassays. The samples were dissolved in methanol for HPLC analysis or in a culture medium for cell experiments.

### Polyphenol characterization in the methanolic extract of *Asplenium trichomanes* L. and *Ceterach officinarum* Willd. fronds by high performance liquid chromatography-tandem mass spectrometry

The quantification of all phytochemicals determined was performed using a modified version of our prior designed assay (35). The Agilent 1290 Infinity series and a Triple Quadrupole 6420 bought from Agilent Technology located in Santa Clara (CA, United States) were used to perform the HPLC-MS/MS and linked to an electrospray ionization (ESI) source that operated in negative and positive ionization modes. Using Optimizer Software, the MS/MS parameters of each standard were optimized by using flow injection analysis (FIA). The isolation of phenolic compounds was obtained by the following methodology: a direct injection of diluted sample (1:5) using gradient elution mode on a Phenomenex Synergi Polar–RP C18 column (250 mm × 4.6 mm, 4 μm) using a mixture of water and methanol as solvents A and B, respectively, both with 0.1% formic acid (starting ratio is 80% A and 20% B). For column protection, a polar RP security guard cartridge preceded the column (4 mm × 3 mm ID). The mobile phase composition was a mixture of the following components: 0–1 min, isocratic condition, 20% B; 1–25 min, 20–85% B; 25–26 min, isocratic condition, 85% B; 26–32 min, 85–20% B. The injection volume was 2 μl, and the flow rate was kept at 0.8 mL/min. The selected temperatures for the column and drying gas in the ionization source were 30 and 350°C, respectively; 12 L/min gas flow rate, 55 psi nebulizer pressure, 4,000 V the capillary voltage, and 2 min the specific time window for each compound (Δ retention time) were the technical specifications of the instrument defined for this assay. The peak areas were integrated for quantitation after detection in the dynamic-multiple reaction monitoring (dynamic-MRM) mode. The transition and collision energies for each compound are provided as Supplementary material. The principle product ion was used for quantification, while the rest of the ions were employed for qualitative analysis.

### Cell culture

The non-tumoral cell lines (mouse fibroblast NIH 3T3 and the spontaneously immortalized human keratinocyte HaCaT cell lines) were used in all *in vitro* experiments. In the case of cytotoxic assay, cell viability was also evaluated by the human tumoral cell lines: cervix epitheloid carcinoma HeLa, Caucasian hepatocyte carcinoma HepG2, Caucasian breast adenocarcinoma MCF-7, and Caucasian lung carcinoma A549 cell lines. Sigma-Aldrich, as a worldwide provider of the European Collection of Authenticated Cell Cultures (ECACC), provided the 3T3 and A549. Whereas Eucellbank (Celltec-Universitat de Barcelona) subminister the HaCaT, HeLa, and MCF-7. Kindly, Dr. Borràs of Experimental Toxicology and Ecotoxicology Platform (UTOX-CERETOX) of Parc Científic of Universitat de Barcelona donated the HepG2 cell line. Cell lines were grown in Dulbecco’s Modified Eagle’s medium (DMEM) supplemented with 10% heat-inactivated fetal bovine serum (FBS), 2 mM L-glutamine, and 100 U/mL:100 U/mL streptomycin–penicillin mixture (10% FBS-DMEM) at 37°C in a 5% carbon dioxide (CO_2_)-humidified incubator. Cells were regularly checked and subsequently subcultured in 75 cm^2^ flasks. When cells reached 80% of confluence in the flask, culture medium was removed, cells were treated with trypsin-EDTA after cleansing with PBS, to obtain a cell suspension. Cell density was determined by staining an aliquot of the cell suspension with the vital dye trypan blue (0.4%), and the final volume was adjusted according to 100 μL of the cell suspension (1 × 10^5^ cells/mL). Which were seeded in 96-well microplates and incubated overnight (37°C and 5% CO_2_).

### Determination of cell viability by the uptake of the Neutral Red dye and thiazolyl blue tetrazolium bromide assays

Cell viabilities were determined by the uptake of the Neutral Red dye (NRU) and thiazolyl blue tetrazolium bromide assays (MTT) methods after treatments.

Some explained adaptations have been applied to the Borenfreund and Puerner protocol for the determination of cell viability by NRU (36). The supernatant was aspirated from each well and 100 μL of NR solution (0.05 mg/mL in DMEM 0% FBS without phenol red) was added after the treatment incubation time. After 3 h, the supernatant was removed by inversion from the plate, and 100 μL of the developer NR solution was added. In the developer solution, the formaldehyde was replaced by an acidic ethanol solution as defined by Riddell et al. (37). The viable cells corresponded to the quantification of the remnant NR, which is the NRU linked to the lysosomes (38). After shaking the plate (5–10 min), the absorbance was obtained at 550 nm, by Tecan Sunrise^®^ microplate reader (Männedorf, Switzerland).

The slight modifications described by Zanette et al. (39) were applied to the MTT assay, based on the experimental protocol of Mosmann (40). A total of 100 μL of an MTT solution (0.5 mg/mL in 0% FBS-DMEM without phenol red) was incorporated in each well following incubation of the plates for at least 3 h in cell culture incubation conditions (37°C and 5% CO_2_). When finished the incubation, the culture medium in all assay wells was substituted for 100 μL of the organic dissolvent dimethyl sulfoxide (DMSO), with the objective to dissolve the formazan crystals (41). The amount of soluble formazan is proportional to the number of cells with optimal mitochondrial activity (42). Before reading the absorbance at 550 nm using a Tecan Sunrise^®^ microplate reader (Männedorf, Switzerland), the homogenization content of each well content was realized by gently shaking the microplate (5 min at 100 rpm/min).

*Cell viability* for NRU and MTT assays was obtained using the following equation:


Cellviability(%)=(Ac⁢o⁢n⁢t⁢r⁢o⁢l-As⁢a⁢m⁢p⁢l⁢eAc⁢o⁢n⁢t⁢r⁢o⁢l)x 100

where *A*_*control*_ is the arc of the absorbance of the control, while *A*_*sample*_ is the absorbance for each sample.

#### Cytotoxicity activity of methanolic extract of *Asplenium trichomanes* L. and *Ceterach officinarum* Willd. fronds in non-tumoral and tumoral cells lines

Each cell lines assayed were treated for 24 h (37°C and 5% CO_2_) with the following increment concentrations: 0.01, 0.1, and 1 mg/mL methanolic fronds extracts in 5% FBS-DMEM. In each plate, the negative controls were the untreated cells (maintained with a culture medium). Cytotoxicity of ATM and COM was evaluated by the NRU and MTT methods.

#### Cytoprotective and cellular repair activity of methanolic extract of *Asplenium trichomanes* L. and *Ceterach officinarum* Willd. fronds in non-tumoral cell lines

For the evaluation of the effect of the application of oxidative stress agent after treatment (cytoprotection) or before extract treatment (cellular repair), hydrogen peroxide was selected as the oxidative stress agent (43).

##### Cytoprotective activity in 3T3 and HaCaT cell lines

Cells were pretreated with the selected methanolic fronds extracts (0.01, 0.1, and 1 mg/mL; 100 μL) dissolved by 5% FBS-DMEM for 24 h following addition of H_2_O_2_ (in 5% FBS-DMEM) at a final concentration 2 mM for 2.5 h. Then, cell viability was determined by NRU and MTT assay. In each microplate, negative and positive controls were included. The positive controls for this assay consist of cells treated with H_2_O_2_ at 2 mM during 2.5 h without previous pretreatment with the extracts.

*Cytoprotective activity* was obtained equation as follows:


Cytoprotectiveactivity(%)

=(C⁢VF⁢e⁢r⁢n⁢e⁢x⁢t⁢r⁢a⁢c⁢t-H⁢2⁢O⁢2-C⁢VH⁢2⁢O⁢2C⁢VF⁢e⁢r⁢n⁢e⁢x⁢t⁢r⁢a⁢c⁢t-H⁢2⁢O⁢2)⁢x⁢ 100

in which the cell viability for each condition is described in the formula where *CV*.

##### Cellular repair activity in 3T3 cell line

The potential cellular repair ability of ferns extracts was evaluated by treating cells with different concentrations of ferns extract (0.01, 0.1, and 1 mg/mL in 5% FBS-DMEM; 100 μL), after induction of oxidative stress with 2 mM H_2_O_2_ 2.5 h. For each independent experiment and plate, negative and positive controls were included. In this case, positive controls consist of the cells with 2 mM H_2_O_2_ treated for 2.5 h but without AAM extract post-treatment. After 24 h incubation, cell viability was assessed by NRU and MTT assays.

*Cellular repair activity* was calculated as follows equation:


Cellularrepairactivity(%)

=(C⁢VF⁢e⁢r⁢n⁢e⁢x⁢t⁢r⁢a⁢c⁢t-H⁢2⁢O⁢2-C⁢VH⁢2⁢O⁢2C⁢VF⁢e⁢r⁢n⁢e⁢x⁢t⁢r⁢a⁢c⁢t-H⁢2⁢O⁢2)⁢x⁢ 100

in which the cell viability for each condition is described in the formula where *CV*.

#### Phototoxicity activity of methanolic extract of *Asplenium trichomanes* L. and *Ceterach officinarum* Willd. fronds in 3T3 and HaCaT cell lines

The potential phototoxic activity of ATM and COM was evaluated following the Organization for Economic Cooperation and Development (OECD) TG 432 (2019) (44) with some adaptions. Briefly, 3T3 and HaCaT cells were seeded at a density to form monolayers at 1 × 10^5^ cells/mL (100 μL) in a 96-well microplate in 10% FBS-DMEM for 24 h. Then, pre-incubated with 0.01, 0.1, and 1 mg/mL methanolic fronds extracts (100 μL). The 0% FBS-DMEM without phenol red was the dissolvent to the samples, while positive and negative controls (cells no treated and treated with chloropromazine, 37.5 μg/mL chlorpromazine, respectively) were dissolved by 0% FBS-DMEM without phenol red. After the 1 h incubation, one plate remains in the dark, and other was exposed to 1.8 J/cm^2^ of ultraviolet A (UVA) light. When finished the irradiation process, the medium was replaced with 100 μL of fresh medium (10% FBS-DMEM) to determine the cell viability after 24 h of incubation by the NRU and MTT colorimetric assays.

Light exposure was performed in a photostability UV chamber (58 cm × 34 cm × 28 cm) with three UVA lamps Actinic BL TL/TL-D/T5 (Philips, 43 V, 352 nm, 15 W) as described by Martínez et al. (45). The dosage and time exposition of cells to UVA light was regularly settled by a photoradiometer Delta OHM provided with a UVA probe (HD2302—Italy). We followed the following equation:


$$E\,\left(\frac{J}{c\,m^{2}}\right)=t\,\left(\text{s}\right)\,x\,P\,\left(\frac{W}{c\,m^{2}}\right)$$

where ultraviolet dose is *E*, represents the time expressed in seconds is *t* and the lamp potency is *P*.

### Intracellular reactive oxygen species induced by H_2_O_2_ of methanolic extract of *Asplenium trichomanes* L. and *Ceterach officinarum* Willd. fronds in 3T3 and HaCaT cell lines

For the ROS assay, we followed the methodology of Ferreira et al. (46). After the pretreatment cells with the different concentrations of the methanolic fronds extracts (0.01, 0.1, and 1 mg/mL) for 24 h, cells were washed twice with PBS, and DCF (100 μM) was applied to each well for 45 min (37°C and 5% CO_2_). To remove the DCF which no penetrated cells, this was removed by washing twice with a cell culture medium, and then H_2_O_2_ (1 and 2 mM) was added into each well to induce oxidative stress. The fluorescence intensity of the oxidized product of DCF was registered ((λ_*excitation*_ 480 nm; λ_*emision*_ 530 nm) at 0, 1, 2, and 3 h by a plate reader ThermoFisher SCIENTIFIC VARIOSKAN LUX (ThermoFisher SCIENTIFIC, Waltham, MA, United States). The fluorescence intensity (FI), which have adimensional units, was used to represent the results. The *FI_*z h Vs 0*__*h*_* was calculated as follows equation:


F⁢l⁢u⁢o⁢r⁢e⁢s⁢c⁢e⁢n⁢c⁢e⁢I⁢n⁢t⁢e⁢n⁢s⁢i⁢t⁢yz⁢h⁢V⁢s⁢ 0⁢h⁢(F⁢Iz⁢h⁢V⁢s⁢ 0⁢h)

=(F⁢Iz⁢h-F⁢I0⁢hF⁢Iz⁢h)⁢x⁢ 100

where *FI*_*z h*_ is the intensity of fluorescence at *z* h (*z* as 1, 2, or 3 h) of incubation and *FI_0__*h*_* is the amount of fluorescence intensity at 0 h.

The *FI* for each specific time was calculated using this formula:


$$F\,I=\frac{F\,l\,u\,o\,r\,e\,n\,c\,e_{480\,n\,m\,\left(e\,x\,c\,i\,t\,a\,t\,i\,o\,n\right)}}{F\,l\,u\,o\,r\,e\,n\,c\,e_{530\,n\,m\,\left(e\,m\,i\,s\,i\,o\,n\right)}}$$

The *ΔROS*, which has adimensional units for FI, was obtained using the following formula:


$$△\,R\,O\,S_{H\,2\,O\,2}\,△\,R\,O\,S_{F\,e\,r\,n\,e\,x\,t\,r\,a\,c\,t\,w\,i\,t\,h\,D\,C\,F-H\,2\,O\,2}-△\,R\,O\,S_{D\,C\,F-H\,2\,O\,2}$$

### Statistical analysis

All experiments were executed in triplicates, and almost three independent experiments were assayed, on different days, except for the cytoprotection ferns extract for HaCaT against 2 mM H_2_O_2_ (2.5 h) MTT for which the results correspond to *n* = 2 experiments. Statistical analysis for MTT cell viability and fluorescence intensity (FI) was performed using GraphPad Prism version 7, San Diego, CA, United States. All data were expressed as mean ± standard error. Activities have been compared using a two-way analysis of variance (ANOVA) by Bonferroni. The results were considered significantly different when *p* ≤ 0.05 (*), *p* ≤ 0.01 (^**^), *p* ≤ 0.001 (^***^), and *p* ≤ 0.0001 (^****^).

## Results

### Phytochemical characterization of methanolic extract of *Asplenium trichomanes* L. and *Ceterach officinarum* Willd. fronds by high performance liquid chromatography-tandem mass spectrometry

Both methanolic extracts present a similar profile of phytochemicals except for neochlorogenic acid, pelargonidin-3-rutinoside, phloridzin, and naringin. Phloridzin was detected for the first time in *A. trichomanes* in low amounts. The phytochemicals neochlorogenic acid and naringin have only been detected in COM, while pelargonidin-3-rutinoside and phloridzin have only been found in ATM. A clearly higher content of phenolic species has been determined in the extract of *C. officinarum* (55,490.94 mg polyphenolic species/kg dry extract) than in the extract of *A. trichomanes* (4,637.30 mg polyphenolic species/kg dry extract), as represented in Table 1. In the *C. officinarum* extract, the content of the majority phytochemical prevails hugely front the rest of all other phytochemicals. This phytochemical is chlorogenic acid and constitutes 78.95% of the total phytochemicals determined in the extract. In the case of the *A. trichomanes* extract, the two major phytochemicals are those that present a higher quantity than the other phytochemicals determined in the extract. These two phytochemicals are flavonols (one type of flavonoids) hyperoside and isoquercitrin, which constitute 54.72% of the determined phytochemicals in the extract.

**TABLE 1:** Content (mg/kg of dry extract) of 38 polyphenolic phytochemicals in the methanolic extract of *Asplenium trichomanes* L. and *Ceterach officinarum* Willd. fronds.

No.	Phytochemicals	Methanolic extract *Asplenium trichomanes* L. fronds (ATM)	Methanolic extract *Ceterach officinarum* Willd. fronds (COM)
	***Phenolic acids***		
1	Gallic acid	5.36	13.14
2	Neochlorogenic acid	n.d.	2,300.25
3	Chlorogenic acid	55.93	43,809.25
4	p-Hydroxybenzoic acid	63.81	31.31
5	3-Hydroxybenzoic acid	n.d.	n.d.
6	Caffeic acid	14.80	112.88
7	Vanillic acid	85.52	213.81
8	Syringic acid	n.d.	n.d.
9	p-Coumaric acid	30.30	67.38
10	Ferulic acid	4.20	1.55
11	3,5-Dicaffeoylquinic acid	1.50	95.85
12	Ellagic acid	67.99	10.10
	***Flavonoids***		
	**(A) Anthocyanins**		
13	Delphinidin-3,5-diglucoside	897.53	1,138.14
14	Delphinidin-3-galactoside	5.85	1.18
15	Cyanidin-3-glucoside	26.13	3.15
16	Petunidin-3-glucoside	n.d.	n.d.
17	Pelargonidin-3-rutinoside	20.79	n.d.
18	Pelargonidin-3-glucoside	n.d.	n.d.
19	Malvidin-3-galactoside	n.d.	n.d.
	**(B) Flavonols**		
20	Rutin	0.98	0.40
21	Isoquercitrin	1,185.02	1,362.95
22	Quercitrin	322.94	2.07
23	Myricetin	n.d.	n.d.
24	Kaempferol-3-glucoside	251.71	263.83
25	Quercetin	26.05	9.85
26	Isorhamnetin	0.06	0.14
27	Hyperoside	1,352.71	1,719.52
28	Kaempferol	52.50	9.56
	**(C) Flavan-3-ols (Flavanols)**		
29	Catechin	n.d.	n.d.
30	Epicatechin	61.43	29.83
31	Procyanidin B2	83.24	39.40
32	Procyanidin A2	1.37	3.15
	**(D) Dihydrochalcones**		
33	Phloridzin	1.30	n.d.
34	Phloretin	n.d.	n.d.
	**(E) Flavanones**		
35	Hesperidin	14.81	15.06
36	Naringin	n.d.	4,234.63
	*Stilbenes*		
37	Resveratrol	n.d.	n.d.
	*Non-phenolic acids*		
38	Trans-cinnamic acid	3.47	2.56
	*Total phenol content*	4,637.30	55,490.94

Fronds analyzed by HPLC-MS/MS (n = 3, RSD% ranged from 1.8 to 6.8%). nd = not detected.

When considering all the phytochemicals determined in HPLC-MS/MS, the different nature of both extracts is increased. Thus, when determining the global nature of the polyphenolic species, it is observed that in the extract of *A. trichomanes*, flavonoids (92.82% total phytochemicals determined) prevail over phenolic acids (7.10% total phytochemicals determined). While in the case of *C. officinarum*, phenolic acids prevail (84.08% total phytochemicals determined) over flavonoids (15.92% total phytochemicals determined).

The phenolic acids 3-hydroxybenzoic acid and syringic acid were not detected in both extracts. Some determined flavonoids represent a low proportion of the total phytochemicals determined for both extracts, as in the case of rutin, isorhamnetin, and procyanidin A2. The flavonoids petunidin-3-glucoside, pelargonidin-3-glucoside, malvidin-3-galactoside, myricetin, catechin, and phloretin were not found in any of the samples. The stilbene resveratrol wasn’t identified either.

### *In vitro* cell assays

The results obtained with the NRU test did not show differences regardless of the conditions tested (data not shown).

#### Cytotoxicity activity of methanolic extract of *Asplenium trichomanes* L. and *Ceterach officinarum* Willd. fronds in non-tumoral and tumoral cell lines

**FIGURE 1:**
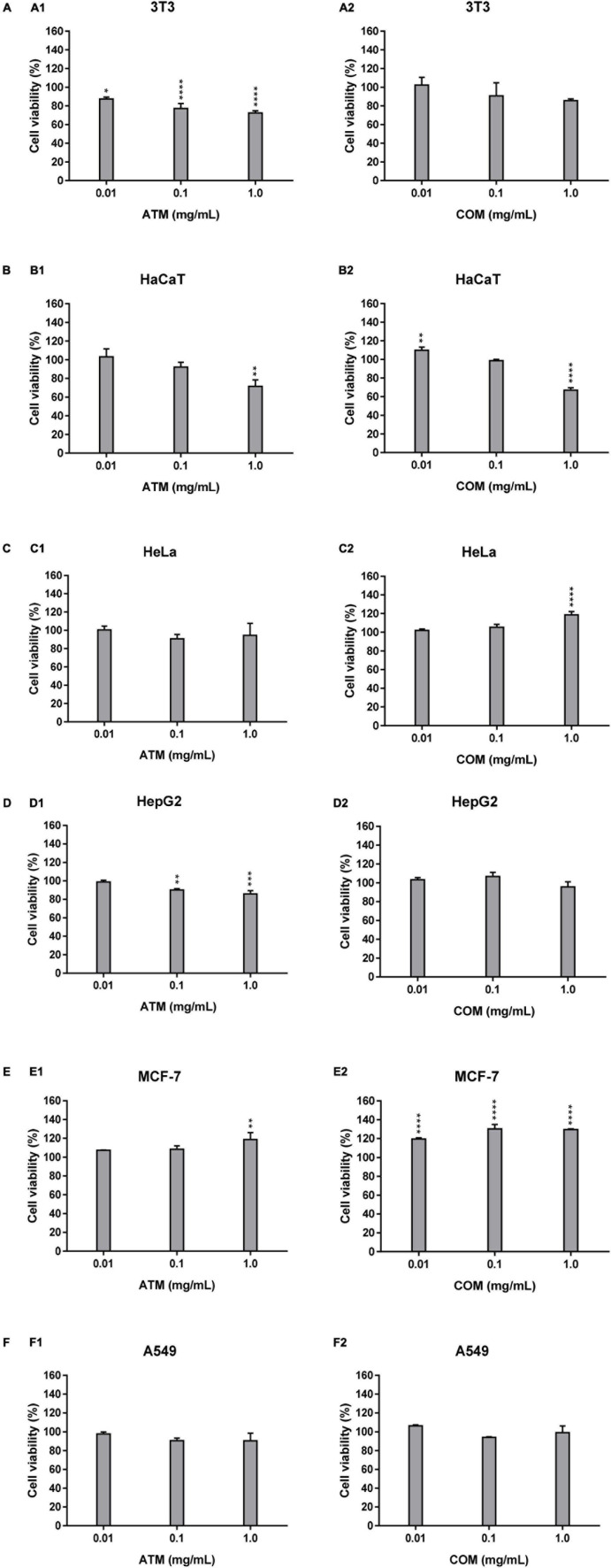
Cytotoxicity activity of ATM (1) and COM (2) in 3T3 **(A)** and HaCaT **(B)**, HeLa **(C)**, HepG2 **(D)**, MCF-7 **(E)**, and A549 **(F)** cell lines by MTT assay and expressed as a percentage of cell viability respect to control cells Results are expressed as mean ± standard error of *n* = 3. Control cells were maintained only with a culture medium. A two-way analysis of variance (ANOVA) and a Bonferroni *post hoc* assay have been performed. Statistical differences were considered as follows: *p ≤ 0.05, **p ≤ 0.01, ***p ≤ 0.001, and ****p ≤ 0.0001 comparison with no treated cells (negative control).

The 3T3 and HaCaT (which are the non-tumoral cell lines assayed), Figures 1A,B, are characterized by the absence of increased cell viability greater than the corresponding negative control. The increment of the ATM concentration leads to a decline in cell viability for 3T3 and HaCaT cell lines, in which ATM is more sensible to 3T3 than HaCaT. While in the case of COM, similar behavior is observed for the two non-tumoral cell lines except at 1 mg/mL COM. The situation in which obtained the lowest cell viability (67.2% cell viability) for the six cell lines assayed. This situation was the only cytotoxic comportment for the six cell lines analyzed as a consequence that cell viability is ≤70%.

In the four human tumoral cell lines, both extracts show a similar behavior only for MCF-7 (Figure 1E). In which cell lines for both extracts present slightly higher cell viability than the negative control. In the case of the remaining tumoral cell lines (HeLa, HepG2, and A549), Figures 1C,D,F, the behavior is similar for each extract. ATM for these three tumoral cell lines, there is a mild decrease in cell viability up to 0.1 mg/mL ATM in which cell viability is stabilized. The COM does not show a clear affectation in the cell viability for HeLa, HepG2, and A549 cell lines at assayed extract concentrations. However, only a slight increase in HeLa cell viability is observed at 1 mg/mL COM. The situation in which there is observed an increment in cell viability less than 20% of the corresponding negative control.

When comparing the behavior of both extracts in non-tumoral versus tumoral cell lines, the only cell line in which a similar behavior has been determined has been in 3T3 and HepG2 for ATM.

#### Cytoprotective activity of methanolic extract of *Asplenium trichomanes* L. and *Ceterach officinarum* Willd. fronds in 3T3 and HaCaT cell lines

Prior to carrying out the cytoprotection assays, the cellular viability of HaCaT was determined for a range of concentrations-time of H_2_O_2_. To define the ideal conditions for the evaluation of the cytoprotective effect of ATM and COM in non-tumoral lines (data not shown). The ideal conditions for the oxidative stress-inducing agent selected were 2 mM H_2_O_2_ for 2.5 h.

**FIGURE 2:**
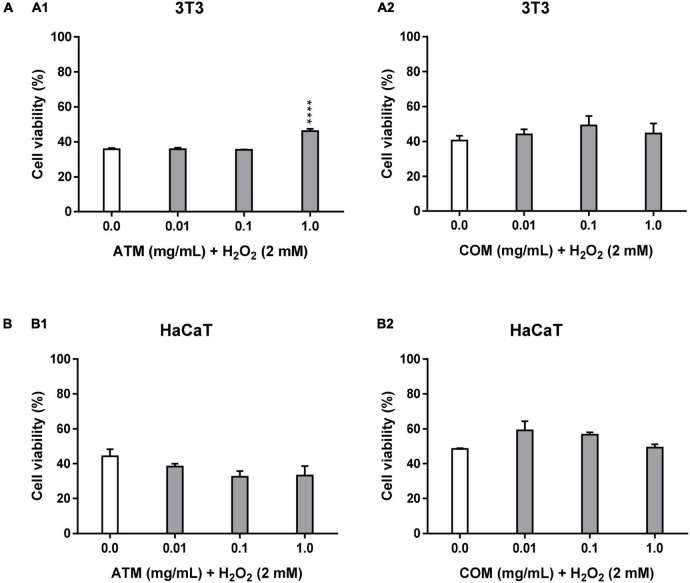
Cytoprotective activity of ATM (1) and COM (2) in 3T3 **(A)** and HaCaT **(B)** cell lines for 2 mM H_2_O_2_ during 2.5 h by MTT assay and expressed as a percentage of cell viability with respect to untreated cells control. H_2_O_2_ cell viability was used as a positive control. Results are expressed as mean ± standard error of *n* = 3 (3T3) and *n* = 2 (HaCaT). A two-way analysis of variance (ANOVA) and a Bonferroni *post hoc* assay have been performed. Statistical differences were considered as follows: ****p ≤ 0.0001 comparison with positive control.

In Table 2, cytoprotective results are shown at 2 mM H_2_O_2_ for 2.5 h. In Figure 2A, cytoprotection of ATM and COM in 3T3 cells against 2 mM H_2_O_2_ for 2.5 h is also demonstrated. Under these same cytoprotection conditions in the case of COM, this extract does not imply statistically significant modifications with respect to the corresponding positive control.

**TABLE 2:** Cytoprotection activity of ATM and COM in 3T3 and HaCaT cell lines for 2 mM H_2_O_2_ during 2.5 h by MTT assay.

Cytoprotection activity of fern extract front 2 mM H_2_O_2_ during 2.5 h by MTT assay				
Fern extract	Concentration of fern (mg/mL)	0.01	0.1	1
ATM	Cytoprotection activity (%)*^a^* in 3T3	0.0	0.0	22.6
	Cytoprotection activity (%)*^a^* in HaCaT	0.0	0.0	0.0
COM	Cytoprotection activity (%)*^a^* in 3T3	8.0	17.6	9.1
	Cytoprotection activity (%)*^a^* in HaCaT	17.8	14.3	1.5

^a^Percentage of cytoprotection activity has been obtained from the following relation [(CV_Fern extract–H2O2_ – CV_H2O2_) / CV_Fern extract–H2O2_] × 100.

Figure 2B shows the behavior of HaCaT in the cytoprotection assay of each of the extracts against 2 mM H_2_O_2_ for 2.5 h. The evolution of cell viability in relation to the increase in ATM concentrations does not present a clear trend. All cell viabilities for ATM concentrations are lower than the corresponding positive control. Nevertheless, COM is the only extract that shows a clary trend in decreasing cell viability with increasing concentration. Without obtaining effective cytoprotection values for COM. No statistically significant cell viability values have been obtained for both extracts in this assay.

#### Cellular repair activity of methanolic extract of *Asplenium trichomanes* L. and *Ceterach officinarum* Willd. fronds in 3T3 cell line

The 3T3 cell viability of the positive controls in ATM and COM in the cellular repair assay (23.8 and 20.4% viability, respectively) is lower than the corresponding ones in the cytoprotection assay (35.7 and 40.5% viability, respectively). This variability is the consequence that the oxidizing agent in the cellular repair assay is applied one day earlier than in the cytoprotective assay (described in methodology).

**FIGURE 3:**
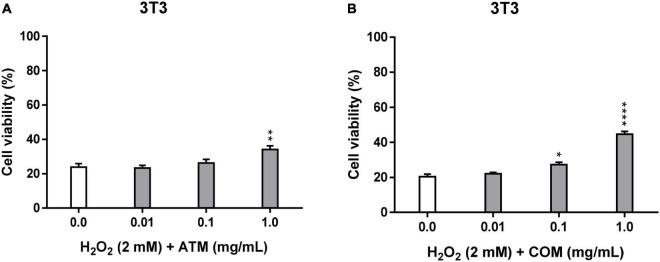
Cellular repair activity of ATM **(A)** and COM **(B)** in 3T3 cell line for 2 mM H_2_O_2_ during 2.5 h by MTT assay and expressed as a percentage of cell viability with respect to untreated cells control. H_2_O_2_ cell viability was used as a positive control. Results are expressed as mean ± standard error of *n* = 3. A two-way analysis of variance (ANOVA) and a Bonferroni *post hoc* assay have been performed. Statistical differences were considered as follows: *p ≤ 0.05, **p ≤ 0.01, and ****p ≤ 0.0001 comparison with positive control.

In the cellular repair assay, due to the absence of a cellular pre-treatment with the extract, which is carried out in the cytoprotection assay, it is expected to obtain lower cell viability than in the corresponding cytoprotection assays. In Figure 3, cellular repair assay in 3T3 for ATM and COM against the conditions of the agent inducing oxidative stress (2 mM H_2_O_2_ for 2.5 h), when compared with the corresponding cell viabilities of the assay of cytoprotection (Figure 2A) except for 1 mg/mL COM. In which cell viability in both cases are similar (Cytoprotective 3T3_1__mg/mL_ _COM_ = 44.5% cell viability; Cellular repair 3T3_1__mg/mL_ _COM_ = 44.6% cell viability).

Both extracts have generally exhibited a greater protective effect in the cellular repair assay than in the corresponding cytoprotective assay. The viability of the samples in the cellular repair assay is always higher than the corresponding positive control in COM (statistically significant values from 0.1 mg/mL COM), while in ATM, it is only from 0.1 mg/mL extract (3T3_0.1__mg/mL_ _ATM_ = 26.1% cell viability), in which the only statistically significant values for ATM are at 1 mg/mL ATM. The trend of the evolution of 3T3 cell viability experienced for ATM in the cytoprotective assay is like that experienced in the cellular repair assay, in which the trend of increasing cell viability as a function of extract concentration is observed clearly from 0.1 to 1 mg/mL ATM. In the case of COM, the evolution of cell viability in the cell repair extract is different from that experienced in the cytoprotective assay (increasing the concentration of extract leads to an increase in cell viability). Result that no clary trend has experienced cell viability with the increasing COM concentration in cellular repair assay.

In the cellular repair assay for both extracts, a percentage of effective cellular repair is obtained only at 1 mg/mL extract. But COM seems clearly superior compared with ATM (Table 3).

**TABLE 3:** Cellular repair activity of ATM and COM in 3T3 cell line for 2 mM H_2_O_2_ during 2.5 h by MTT assay.

Oxidative agent conditions	Concentration of fern (mg/mL)	0.01	0.1	1
2 mM H_2_O_2_ during 2.5 h	3T3 cellular repair activity (%)*^a^* by ATM	0.0	9.0	30.2
	HaCaT cellular repair activity (%)*^a^* by COM	7.5	24.9	54.2

^a^Percentage of cellular repair activity has been obtained from the following relation [(CV_Fern extract–H2O2_ – CV_H2O2_) / CV_Fern extract–H2O2_] × 100.

#### Phototoxicity activity of methanolic extract of *Asplenium trichomanes* L. and *Ceterach officinarum* Willd. fronds in 3T3 and HaCaT cell lines

Irradiated DMEM control versus non-irradiated DMEM control gates a decrease of around 30% cell viability for 3T3 and HaCaT cell lines (Table 4). One possible bias of the low ratio of this control has been eliminated, obtaining the corresponding viabilities with respect to the non-irradiated DMEM (in the graphic representation of the phototoxicity test, the cell viability is represented as a function of the non-irradiated DMEM for the non-irradiated ones and the irradiated DMEM for irradiated).

**TABLE 4:** Comparison of cell viability of irradiated with non-irradiate controls*^a^*.

Cell line	DMEM (%)	CPZ (%)
3T3	68.0	34.8
HaCaT	73.4	16.4

^a^Expressed as the percentage of cell viability of the irradiated control in relation to the corresponding non-irradiated.

The positive control shows a high phototoxic effect of irradiated chlorpromazine compared to the corresponding non-irradiated situation. Obtaining a greater sensitivity of the oxidative stress inducing agent in HaCaT than in 3T3, which is the contrast obtained in the positive cytoprotection control at 2 mM H_2_O_2_ for 2.5 h.

**FIGURE 4:**
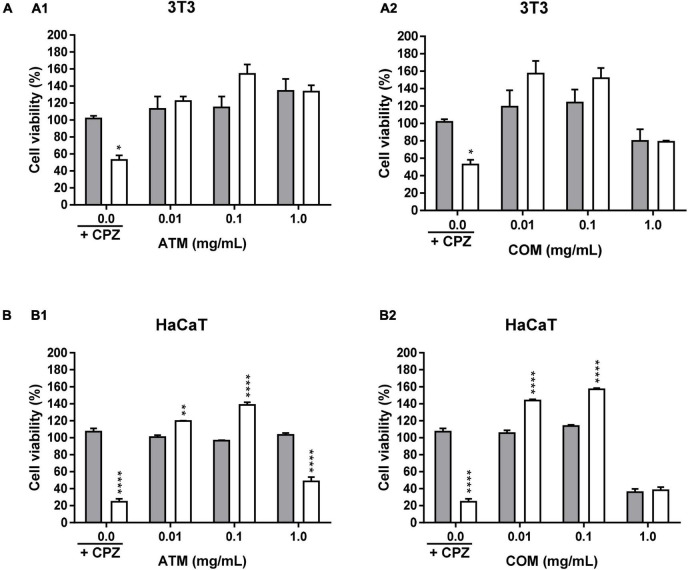
Phototoxicity activity of ATM (1) and COM (2) in 3T3 **(A)** and HaCaT **(B)** cell lines by MTT assay and expressed as a percentage of cell viability with respect to the correspondent control cells Chlorpromazine cell viability was used as positive control. Gray columns correspond to cells nonexposed to UVA light and white columns correspond to cells exposed to 1.8 J/cm^2^ of UVA light. Results are expressed as mean ± standard error of *n* = 3. A two-way analysis of variance (ANOVA) and a Bonferroni *post hoc* assay have been performed. Statistical differences were considered as follows: *p ≤ 0.05, **p ≤ 0.01, and ****p ≤ 0.0001 comparison with the equivalent nonirradiated condition homologue.

Figure 4 represents the phototoxicity test at 1.8 J/cm^2^ for 3T3 and HaCaT using MTT for ATM and COM. In none of the concentrations tested for both extracts for 3T3 have phototoxic behavior been obtained (Figure 4A). Presenting both extracts a similar behavior to 0.01 and 0.1 mg/mL extract with higher cell viability in the irradiated homologue than in the non-irradiated one. The fact that by presenting an increase in the cell viability of the irradiated compared to the non-irradiated one ≥20% in ATM at 0.1 mg/mL extract and in COM at 0.01 and 0.1 mg/mL extract, a possible photoprotective effect is considered. At 1 mg/mL COM, no irradiated decrease in cell viability is manifested in comparison to CPZ no irradiated. A situation that confirms the previous cytotoxic comportment of this extract in 3T3 at the highest concentration assayed.

Figure 4B represents the phototoxicity test at 1.8 J/cm^2^ for the ATM and COM extracts in HaCaT by MTT. At 0.01 and 0.1 mg/mL extract, the same behavior is experienced as in the corresponding situation as in 3T3. Wherein the viability between homologues is higher in the irradiated compared to the corresponding non-irradiated. However, in 1 mg/mL COM, the effects previously observed in cytotoxicity assay prevail. While ATM, despite not observing a significant decrease in cell viability in the non-irradiated 1 mg/mL extract (it is not a cytotoxic extract at assayed conditions), in the corresponding irradiated situation the extract does not have the ability to reverse the photosensitizing effect induced by UVA.

#### Intracellular ROS induced by H_2_O_2_ of methanolic extract of *Asplenium trichomanes* L. and *Ceterach officinarum* Willd. fronds in 3T3 and HaCaT cell lines

The ROS value does not undergo significant modifications over time. Rather, time only slightly increases ROS values without modifying the profile experienced in each of the times analyzed (1, 2, and 3 h). The reason why only ROS is represented at 2 h (ROS at 1 h and 3 h not represented).

The ROS value for both 3T3 positive controls (at 1 and 2 mM H_2_O_2_; for each concentration of H_2_O_2_ are the same positive controls for both extracts) is higher than the corresponding HaCaT values (ROS positive control for 1 mM H_2_O_2_: ROS_1__mM H2O2 for 3T3_ = 168.0 and ROS_1__mM H2O2 for HaCaT_ = 82.3; ROS positive control for 2 mM H_2_O_2_: ROS_2__mM H2O2 for 3T3_ = 153.9 and ROS_2__mM H2O2 for HaCaT_ = 110.0). However, the fact that in 3T3 higher ROS has been determined in 1 mM H_2_O_2_ than in 2 mM H_2_O_2_ is due to possible cell death at 2 mM H_2_O_2_. This is in line with the low cell viability of the positive controls in the cytoprotection at 2 mM H_2_O_2_ for 2.5 h assays in 3T3 compared to the corresponding ones in HaCaT. Because protective activity and ROS values are inversely related (less protective activity of cell line entails higher ROS values).

**FIGURE 5:**
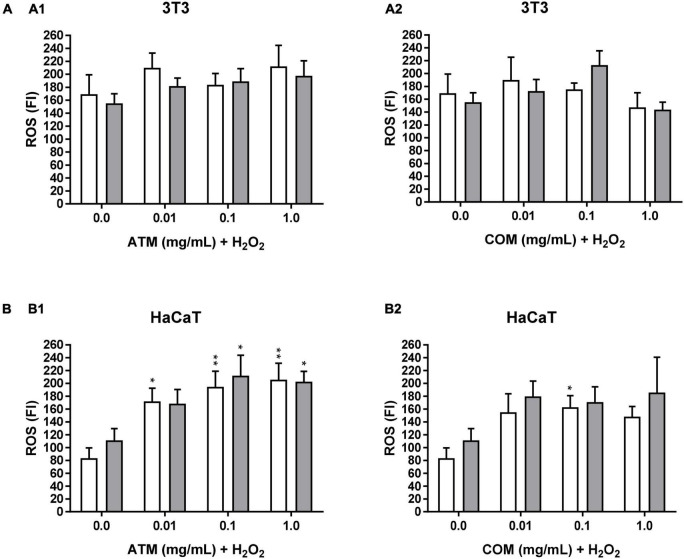
Intracellular ROS induced by 1 and 2 mM H_2_O_2_ for 2 h treatment with ATM (1) and COM (2) in 3T3 (**A)** and HaCaT **(B)** cells H_2_O_2_ cell viability was used as a positive control. White columns correspond to 1 mM H_2_O_2_, and gray columns correspond to 2 mM H_2_O_2_. Results are expressed as mean ± standard error of *n* = 3. A two-way analysis of variance (ANOVA) and a Bonferroni *post hoc* assay have been performed. Statistical differences were considered as follows: *p ≤ 0.05 and **p ≤ 0.01 comparison with the corresponding positive control.

In Figure 5A, ROS assay in 3T3 for ATM and COM, a different evolution of ROS in relation to the increase in the concentration of each of the extracts is observed by both conditions of oxidative stress (1 and 2 mM H_2_O_2_). In all the tested conditions, the ROS value is always higher in ATM with respect to the corresponding COM except for 0.1 mg/mL COM at 2 mM H_2_O_2_. In the ATM extract, no clear trend is observed. In the case of COM, an increase in ROS to 0.01 and 0.1 mg/mL extract at 2 mM H_2_O_2_ stands out, possibly due to the increment of oxidative stress. Finally, at 1 mg/mL COM for both concentrations of H_2_O_2_ tested, the cytotoxic effects of the COM prevail, as observed in the cytotoxicity assay, producing decreases in the increase of ROS due to cell death itself.

In Figure 5B, ROS assay in HaCaT for ATM and COM, the same trend is determined for both H_2_O_2_ conditions applied in each extract assay. Where ROS increases with respect to the corresponding positive controls. Causing the majority of ROS increases to be positive (Table 5). A clear trend has been observed that increasing the ATM concentration carries an increase in ROS value. While in the case of COM, there is a decline in ROS from 0.1 mg/mL extract in both oxidative stress conditions assayed. This coincides is the case in the corresponding cytotoxicity assays in Figure 1B (cytotoxicity of both extracts in HaCaT).

**TABLE 5:** Intracellular ΔROS*^a^* induced by 1 and 2 mM H_2_O_2_ for 2 h with respect to 0 h at different concentrations of ATM and COM in 3T3 and HaCaT.

Cell line	3T3			HaCaT		
Concentration of extract (mg/mL)	0.01	0.1	1	0.01	0.1	1
1 mM H_2_O_2_ for ATM	40.6	14.4	42.9	88.3	111.2	122.1
1 mM H_2_O_2_ for COM	20.7	6.0	–21.9	71.3	79.3	64.5
2 mM H_2_O_2_ for ATM	26.6	34.0	42.7	57.2	100.7	91.4
2 mM H_2_O_2_ for COM	17.7	57.9	−11.5	68.4	59.5	74.5

^a^Intracellular ΔROS cytoprotective activity has been obtained from the following relation ROS_Fern extract with DCF–H2O2_ – ROS_DCF–H2O2_ expressed as fluorescence intensity (FI).

## Discussion

Although ferns have been poorly studied from a phytochemical and nutritional approach, fronds are the main part of the ferns used to make medicinal preparations by the *Malayalis* in Kolli hills, India (9). The election of the extraction solvent is a relevant aspect in the phytochemical determination of plant drugs. Selectivity (based on polarity target phytochemical), safety, neutral, and easy to separate from the rest of no target phytochemicals, low viscosity, low boiling temperature, and economical are the criteria for selecting the extraction solvent (47). Currently, most plant matrices are obtained from organic solvents compared to other solvents such as green solvents (environmentally friendly). For the *in vitro* studies that we have carried out on *A. trichomanes* and *C. officinarum* extracts, we have used the methanolic extract of their fronds while the correspondence hexane extract was discarded due to the lower presence of total polyphenol content (TPC) than the corresponding methanolic extract in a previous study (34).

In the Russian Far East, there are a minimum of 13 fronds of edible fern species, where *P. aquilinum* predominates (11). The degree of maturation of the fronds (young fronds, as known fern shots or fiddleheads, or mature fronds) and the way in which the fronds are preserved (by freezing, canning, salting, or drying) are important to determine the potential toxicity of edible ferns. For example, different government dossiers regard the safeguard of public health against *P. aquilinum* to whose toxins affect human populations and animals. The main toxin that triggers the carcinogenesis of *P. aquilinum* is ptaquiloside (an illudane, a subclass of sesquiterpenes) (48, 49). Currently, ferns are not incorporated into the diet of European countries. However, the publication of nutritional properties of plant matrices of ferns may be an incentive to incorporate ferns in European diets (14).

We have considered the methanolic extracts of the fronds of *A. trichomanes* and *C. officinarum* as potential reservoirs of phytochemicals. Derived that other *Aspleniaceae* species have been described as reservoirs of phytochemicals. Such as the ferns *Asplenium adiantum-nigrum* L. (*Aspleniaceae*) and *Asplenium ruta-muraria* L. (*Aspleniaceae*) (50), of which the methanolic extract of the fronds of both ferns is the part with the highest total phenol content (TPC) and total flavonoid content (TFC). And specifically, the content of flavonoids in the fronds is relevant due to one of the functions of the flavonoids in the fronds, which are the neutralization of UV radiation and ROS (51). In the phytocharacterization by HPLC-MS/MS of ATM fronds, a greater quantity of flavonoids, especially flavonols related to kaempferol, was obtained, as Dall’Acqua et al. (52) described. In our phytocharacterization, the main phytochemical isolated from ATM has been hyperoside, another flavonol. We have also isolated kaempferol and kaempferol glycoside (kaempferol-3-glucoside). Resulting that flavonols are the main polyphenolic species determined in ATM, representing 68.8% of the total phytochemicals determined by HPLC-MS/MS. In the case of COM, flavonoids are minor phytochemicals detected by HPLC-MS/MS compared to phenolic acids. These results are equivalent to that obtained by Zivkovic et al. (53). In contrast to Zivkovic et al. (53), we have detected fractions of rutin in the fronds of *C. officinarum*. In this study, chlorogenic acid is the main phytochemical determined in COM, while the other cinnamic acids such as caffeic acid are only detected in traces. This observation has already been previously reported by Tomou et al. (28). Phytocharacterization by HPLC-MS/MS of both extracts coincides with the nature of the phytochemicals determined by TLC in a previous study (34).

Durdevic et al. (54) determined that the content of phenolic species in the ethylacetate extracts of *A. trichomanes* and *C. officinarum* is higher in the frond than in the correspondence rhizome. And the predominance of aglycone phytochemicals (no sugar phytochemicals) than glycone phytochemicals (sugar phytochemicals) in the ethylacetate frond extract. We have determined a higher amount of TPC in the methanolic extract of the fronds of *C. officinarum* than in the methanolic extract of the fronds of *A. trichomanes*, as described by Durdevic et al. (54).

Currently, there is a great discrepancy in the cytotoxicity of ferns, derived from the fact that they are a widely diversified taxon. However, in the cytotoxic determination of methanolic extracts, different species of ferns have concluded greater cytotoxicity in the frond than the corresponding rhizome (55). For this reason, it is important that cytotoxicity assays have been performed in a wide range of concentrations. The most common assays to determine cell viability are the reduction in the tetrazole salt or MTT, the uptake of the Neutral Red dye (NRU), or the release of lactate dehydrogenase among others (42). The measurements of most of these techniques are end point and present advantages and disadvantages. In this sense, MTT is usually the method of choice in different cytotoxic studies (38), because it has proven to be valid with different cell lines and is relatively straightforward and useful when conditions are optimized. Moreover, the MTT assay has been used to characterize the cytotoxic profile of different plant extracts including ferns (34). Actually, MTT is regarded as a gold standard of initial cytotoxicity assays as it is highly sensitive and a high-throughput screening assay together with its low economic cost (56). Nevertheless, the NRU test failed to be sensitive in our case. We are also aware that in our research there may be potential interferences of the extracts in the assays by colorimetric methods. It is described that some phytochemicals can interact with MTT producing false-positive cell viabilities (57). For this reason, the absence of interference has been verified by the MTT assay for the *in vitro* concentrations of the extracts tested. Petkov et al. 2021 (58) analyzed the cytotoxicity of methanolic fronds extracts of three *Aspleniaceae* ferns by MTT, of which two were *A. trichomanes* and *C. officinarum*. The comparison of our cytotoxicity results with the results of Petkov et al. 2021 (58) is relevant due to the same methodology for obtaining the frond extract (frond methanolic extract) and for determination of cell viability (MTT assay) for *A. trichomanes* and *C. officinarum*. In the initial toxicity studies of the pharmaceutical industry, assays with a high sensitivity against cytotoxicity with reasonable cost are used, in which various hepatic cell lines are generally used, such as HepG2 (59). The absence of cytotoxicity of the extracts in HepG2 confirms the safety of these extracts.

A diet rich in antioxidants and polyphenols contributes to reducing the risk of diseases resulting from oxidative damage. However, the employment of synthetic antioxidants, as in the case of butylated hydroquinone, in recent years, has been decreased for safety reasons (51). For this reason, investigations of new plant matrices with a high content of antioxidants have increased (60). Despite the low number of studies on ferns, the methanolic extract of their fronds is considered plant parts with a high amount of antioxidant phytochemicals (61, 62). Currently, the extracts of *Polypodiaceae* ferns such as *Polypodium leucotomos* (aqueous extract) and *Polypodium vulgare* L. (methanolic extract) have been reported as antioxidant and cytoprotective agents *in vitro* (63, 64). However, few ferns of the *Aspleniaceae* family, despite being the main ferns in Europe, have been studied as plant matrices for the contribution of antioxidant phytochemicals (14). For example, in the methanolic extract of *A. adiantum-nigrum* and *C. officinarum* (both species are *Aspleniaceae* family), mangiferins and mangiferin-related phytochemicals have also been isolated among other polyphenolic phytochemicals (50, 53). This fact demonstrates the potential of the *Aspleniaceae* as a reservoir of polyphenolic phytochemicals.

## Conclusion

The methanolic extracts of the fronds of the two main species of ferns from the Prades mountains, *A. trichomanes* and *C. officinarum*, present interesting phytochemicals of different nature without being cytotoxic in the mouse fibroblast 3T3, human keratinocyte HaCaT, cervical human cancer HeLa, liver human cancer HepG2, breast human cancer MCF-7, and lung human cancer A549 cell lines at the assayed concentrations. This fact provides new evidence for considering certain ferns of the *Aspleniaceae* family as plant matrices for the extraction of phytochemicals of pharmaceutical or nutritional interest.

## Data availability statement

The raw data supporting the conclusions of this article will be made available by the authors, without undue reservation.

## Author contributions

MM and VL: conceptualization, writing-review and editing, and supervision. AF, MM, and FM: methodology. AF, FM, GC, and MM: analysis. AF, MM, and VL: investigation. MM, MV, and VL: resources. AF: writing—original draft preparation. MM and MV: funding acquisition. All authors have read and agreed to the published version of the manuscript.

## Figures and Tables

**Table:** 

3T3 = NIH 3T3	NIH 3T3 mouse fibroblast cell line
A549	Human Caucasian lung carcinoma
ATM	Methanolic extract of *Asplenium trichomanes* L. fronds
COM	Methanolic extract of *Ceterach officinarum* L. fronds
CO_2_	Carbon dioxide
CPZ	Chlorpromazine hydrochloride
CV	Cell Viability
E	Ultraviolet dose
DCF	2,7-dichlorodihydrofluorescein diacetate
DMEM	Dulbecco’s Modified Eagle’s Medium
DMSO	Dimethyl sulfoxide
ECACC	European Collection of Authenticated Cell Cultures
EDTA	Ethylenediaminetetraacetic acid
FBS	Fetal Bovine Serum
FI	Fluorescence Intensity
HaCaT	Spontaneously immortalized human keratinocyte cell line
HeLa	Human cervix epitheloid carcinoma
HepG2	Human Caucasian hepatocyte carcinoma
HPLC-MS/MS	High performance liquid chromatography-tandem mass spectrometry
H_2_O_2_	Hydrogen peroxide
MCF-7	Human Caucasian breast adenocarcinoma
MTT	2,5-diphenyl-3-(4,5-dimethyl-2-thiazolyl) tetrazolium bromide or thiazolyl blue tetrazolium bromide
NR	Neutral Red
NRU	Neutral Red Uptake or uptake of the Neutral Red dye
OECD	Organisation for Economic Cooperation and Development
PBS	Phosphate Buffered Saline
PLE	Aqueous extract of *Polypodium leucotomos* fronds
ROS	Reactive Oxygen Species
TLC	Thin Layer Chromatography
TFC	Total Flavonoids Content
TPC	Total Phenol/Polyphenol Content
UV	Ultraviolet
UVA	Ultraviolet A
