# Supplementary material for: Exploring wild Aspleniaceae ferns as safety sources of polyphenols: The case of Asplenium trichomanes L. and Ceterach officinarum Willd
Source: Front Nutr. 2022 Sep 12;9:994215. doi: 10.3389/fnut.2022.994215 (PMC9511145; doi:10.3389/fnut.2022.994215)
Supplement: Supplementary file 1 [file Table_1.DOCX]

**Supplementary material S1**

HPLC–MS/MS acquisition parameters (dynamic-MRM mode) used for the analysis of the 38 marker compounds.

| No. | Compounds | Precursor ion, *m/z* | Product ion, *m/z* | Fragm-entor, V | Collision energy, V | Polarity |
| --- | --- | --- | --- | --- | --- | --- |
| 1 | Gallic acid | 169 | 125.2^*^ | 97 | 12 | Negative |
| 2 | Neochlorogenic acid | 353 | 191.2^*^, 179 | 82 | 12, 12 | Negative |
| 3 | Delphinidin-3-galactoside | 465.01 | 303^*^ | 121 | 20 | Positive |
| 4 | (+)-Catechin | 289 | 245.2^*^,109.2 | 131 | 8, 20 | Negative |
| 5 | Procyanidin B2 | 576.99 | 576.99^*^, 321.2 | 160 | 0, 32 | Negative |
| 6 | Chlorogenic acid | 353 | 191.2^*^, 127.5 | 82 | 12, 20 | Negative |
| 7 | *p*-Hydroxybenzoic acid | 137 | 93.2^*^ | 92 | 16 | Negative |
| 8 | (-)-Epicatechin | 289 | 245.1^*^, 109.1 | 126 | 8, 20 | Negative |
| 9 | Cyanidin-3-glucoside | 449 | 287.3^*^, 255.6 | 121 | 20, 20 | Positive |
| 10 | Petunidin-3-glucoside | 479.01 | 317^*^, 302 | 121 | 20, 44 | Positive |
| 11 | 3-Hydroxybenzoic acid | 137 | 93.2^*^ | 88 | 8 | Negative |
| 12 | Caffeic acid | 179 | 135.2^*^, 134.1 | 92 | 12, 24 | Negative |
| 13 | Vanillic acid | 167 | 152.4^*^, 108.1 | 88 | 12, 20 | Negative |
| 14 | Pelargonidin-3-glucoside | 433.01 | 271^*^, 121 | 116 | 24, 50 | Positive |
| 15 | Pelagonidin-3-rutinoside | 579.01 | 271^*^ | 145 | 32 | Positive |
| 16 | Malvidin-3-galactoside | 493.01 | 331^*^, 315.1 | 121 | 20, 50 | Positive |
| 17 | Syringic acid | 196.9 | 182.2^*^, 121.2 | 93 | 8, 12 | Negative |
| 18 | Procyanidin A2 | 575 | 575^*^, 285 | 170 | 0, 20 | Negative |
| 19 | *p*-Coumaric acid | 163 | 119.2^*^, 93.2 | 83 | 12, 36 | Negative |
| 20 | Ferulic acid | 193 | 134.2^*^, 131.6 | 83 | 12, 8 | Negative |
| 21 | 3,5-Dicaffeoylquinic acid | 514.9 | 353.1^*^, 191 | 117 | 8, 28 | Negative |
| 22 | Rutin | 609 | 300.2^*^, 271.2 | 170 | 32, 50 | Negative |
| 23 | Hyperoside | 465.01 | 303^*^, 61.1 | 97 | 8, 50 | Positive |
| 24 | Isoquercitrin | 463 | 271.2^*^, 300.2 | 155 | 44, 24 | Negative |
| 25 | Delphinidin-3,5-diglucoside | 462.9 | 300.1^*^ | 165 | 24 | Negative |
| 26 | Phloridzin | 435.39 | 273^*^, 167 | 155 | 8, 28 | Negative |
| 27 | Quercitrin | 446.99 | 300.2^*^, 301.2 | 160 | 24, 16 | Negative |
| 28 | Myricetin | 316.99 | 179.1^*^, 182 | 150 | 16, 24 | Negative |
| 29 | Naringin | 578.99 | 271.3^*^, 151.3 | 170 | 32, 44 | Negative |
| 30 | Kaempferol-3-glucoside | 447 | 284.2^*^, 255.2 | 170 | 24, 40 | Negative |
| 31 | Resveratrol | 227 | 185^*^ | 60 | 40 | Negative |
| 32 | Hesperidin | 611.01 | 303^*^, 334.8 | 112 | 20, 12 | Positive |
| 33 | Ellagic acid | 301 | 301^*^, 229 | 170 | 0, 24 | Negative |
| 34 | Trans-cinnamic acid | 149 | 131.2^*^ | 120 | 30 | Negative |
| 35 | Quercetin | 300.99 | 151.2^*^, 179.2 | 145 | 16, 12 | Negative |
| 36 | Phloretin | 272.99 | 167^*^, 123 | 116 | 8, 20 | Negative |
| 37 | Kaempferol | 287.01 | 153^*^, 69.1 | 60 | 36, 50 | Positive |
| 38 | Isorhamnetin | 314.99 | 300.2^*^, 196.1 | 145 | 16, 4 | Negative |

^*^ These product ions were used for quantification.
