# Supplementary material for: Exploring wild Aspleniaceae ferns as safety sources of polyphenols: The case of Asplenium trichomanes L. and Ceterach officinarum Willd
Source: Front Nutr. 2022 Sep 12;9:994215. doi: 10.3389/fnut.2022.994215 (PMC9511145; doi:10.3389/fnut.2022.994215)
Supplement: Supplementary file 2 [file Presentation_1.pptx]

## Slide 1
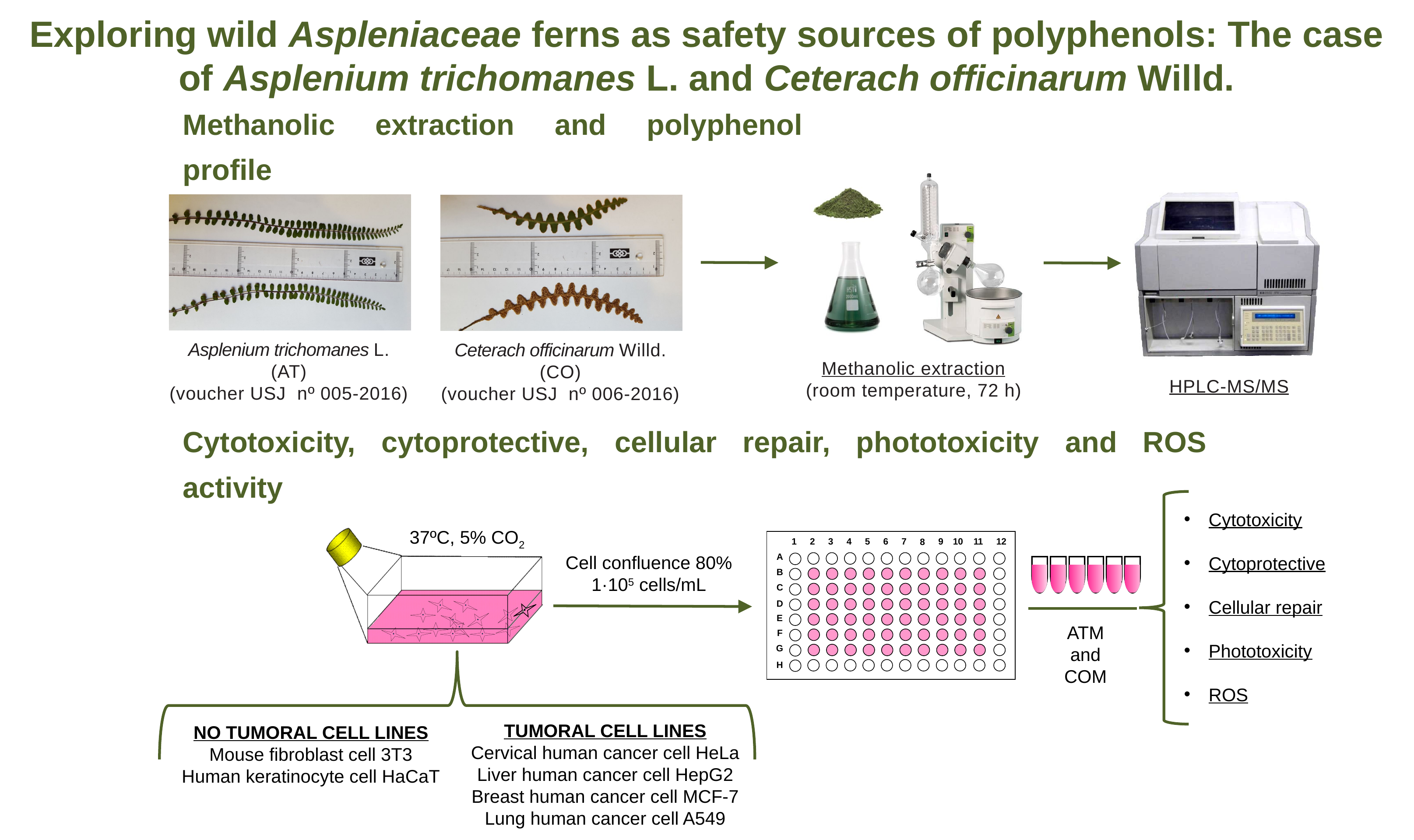

Exploring wild Aspleniaceae ferns as safety sources of polyphenols: The case of Asplenium trichomanes L. and Ceterach officinarum Willd.
Methanolic extraction and polyphenol profile
Asplenium trichomanes L.
(AT)
(voucher USJ nº 005-2016)
Ceterach officinarum Willd.
(CO)
(voucher USJ nº 006-2016)
Methanolic extraction
(room temperature, 72 h)
HPLC-MS/MS
Cytotoxicity, cytoprotective, cellular repair, phototoxicity and ROS activity
Cytotoxicity
Cytoprotective
Cellular repair
Phototoxicity
ROS
37ºC, 5% CO2
1
2
3
4
5
6
7
9
10
11
12
8
A
B
C
D
E
F
G
H
Cell confluence 80%
1·105 cells/mL
ATM
and
COM
TUMORAL CELL LINES
Cervical human cancer cell HeLa
Liver human cancer cell HepG2
Breast human cancer cell MCF-7
Lung human cancer cell A549
NO TUMORAL CELL LINES
Mouse fibroblast cell 3T3
Human keratinocyte cell HaCaT
